# Authentication of *Apis cerana* Honey and *Apis mellifera* Honey Based on Major Royal Jelly Protein 2 Gene

**DOI:** 10.3390/molecules24020289

**Published:** 2019-01-14

**Authors:** Yan-Zheng Zhang, Shuai Wang, Yi-Fan Chen, Yu-Qi Wu, Jing Tian, Juan-Juan Si, Cui-Ping Zhang, Huo-Qing Zheng, Fu-Liang Hu

**Affiliations:** College of Animal Science, Zhejiang University, Hangzhou 310058, China; 21417023@zju.edu.cn (Y.-Z.Z.); troywang0420@foxmail.com (S.W.); cgxllx@zju.edu.cn (Y.-F.C.); hzhzwuyuqi@163.com (Y.-Q.W.); tj_1224@126.com (J.T.); 18868108834@163.com (J.-J.S.); lgzcplyx@aliyun.com (C.-P.Z.); hqzheng@zju.edu.cn (H.-Q.Z.)

**Keywords:** honey, *Apis cerana*, *Apis mellifera*, authentication, *MRJP2* gene

## Abstract

In Asia, honey is mainly produced by *Apis mellifera* and *Apis cerana*. However, the price of *A. cerana* honey is usually much higher than *A. mellifera* honey. Seeing considerable profits, some dishonest companies and beekeepers mislabel *A. mellifera* honey as *A. cerana* honey or incorporate *A. mellifera* honey into *A. cerana* honey. In the present study, we developed methods to discriminate *A. cerana* honey from *A. mellifera* honey based on the *MRJP2* (major royal jelly protein 2) gene. Two pairs of species-specific primers were designed. The amplification products of *A. cerana* and *A. mellifera* were 212 and 560 bp, respectively. As little as one percent incorporation of *A. mellifera* honey in the mixture can be detected by duplex PCR. Additionally, another method based on the melt curve analysis using the same primers was also developed, allowing a rapid discrimination of real-time PCR product of different species. Our study shows that the entomological authentication of honey samples can be identified by nuclear genes other than mitochondrial genes and this extends the possibility of gene selection in identification. The authentication system we proposed could be a useful tool for discriminating *A. cerana* honey from *A. mellifera* honey.

## 1. Introduction

As a famous natural health product, honey is widely consumed around the world. Unfortunately, honey is one of the most frequently counterfeited natural health products [1]. The botanical, geographical, and entomological origin have great influence on the composition and price of honey. Nowadays, honey frauds include adulteration of honey with sugar and syrups and mislabeling of floral origin and bee species [2,3,4,5].

With a growing demand for local and traditional products recently, the entomological origin of honey has received considerable attention in honey quality control. Honey bees are mainly classified into three major clusters: cavity-nesting bees (*A. mellifera*, *A. cerana*, *A. koschevnikovi*, *A. nuluensis*, and *A. nigrocincta*), giant bees (*A. dorsata*, *A. laboriosa* and *A. binghami*) and dwarf bees (*A. Xorea* and *A. andreniformis*) [6]. Among these species, the western honeybee *A. mellifera* and the eastern honeybee *A. cerana* are two dominant species for honey production [7]. Because *A. mellifera* is more suitable for modern breeding and way more productive, it has been introduced into many *A. cerana* habitats, such as China, South Korea, and some southeast Asian countries [8,9]. In China and South Korea, there have been intense competition between the two species, resulting in the decline of *A. cerana* populations [10,11]. In Asian markets, the price of *A. cerana* honey is usually three to five times higher than that of *A. mellifera* honey due to limited productivity and local consumer preferences. Thus, some dishonest honey sales companies and beekeepers may mislabel *A. mellifera* honey as *A. cerana* honey or incorporate *A. mellifera* honey into *A. cerana* honey [7,10]. Investigations on distinguishing the two kinds of honey are rare, it is necessary and urgent to develop analytical methodologies that can differentiate *A. cerana* honey from *A. mellifera* honey to protect the essential interests of consumers and honest *A. cerana* beekeepers.

There are three main types of methods have been developed to identify the entomological origin of honey. The protein-based methods were frequently used before. Deug-Chan et al. [12] used sodium dodecyl sulfate polyacrylamide gel electrophoresis (SDS-PAGE) for determining the entomological origin of honeys produced by Korean native bees and by European bees. They found that there were differences in the protein SDS-PAGE profiles of these two kinds of honey. Sera et al. [13] found that *MRJP1* (major royal jelly protein 1)have different molecular weights and surface structures in *A. cerana* and *A. mellifera* honeys, based on which they successfully identified the entomological origin of those two honeys. Ramónsierra et al. [14] used SDS-PAGE to analyze the protein profiles of stingless bee honey and *A. mellifera* honey, they suggested that protein SDS-PAGE might be an effective method to establish the entomological origin of stingless bee honeys. In addition, a protein-based method was also proposed by Zhang et al. [15] to detect *A. cerana* honey adulterated with *A. mellifera* honey. Beeswax components-based methods were also proposed to identify the entomological origin of honey. Zuccato et al. [16] have analyzed the chloroform extracts spectra of honey produced by stingless bee and *A. mellifera* using ^1^H-NMR coupled with chemometrics. They achieved a successful entomological discrimination between these honeys, and found some diagnostic entomological markers were derived from beeswax. Zhang et al. [15] found that 17-Pentatriacontene and Hentriacontane were the characteristic constituents of *A. cerana* honey and *A. mellifera* honey, respectively, and their work showed that these two characteristic ingredients came from their own beeswax.

Recently, DNA-based methods were considered as the most suitable tools for species identification in animal products and processed foods [17]. Compared to other methods mentioned above, DNA-based methods are considered to be quicker and more accurate, and have advantages of stability and ubiquity in all cell types. Bee DNA exists in honey, and thus can be used to identify the entomological origin of honey. Prosser and Hebert [18] described a protocol that employed DNA metabarcoding of three gene regions (*ITS2, rbcLa*, and *COI*) to provide a cheap tool to deliver information on the botanical and entomological origins of honey at the same time. Recently, two studies [7,19] have performed identification of *A. cerana* honey and *A. mellifera* honey by genetic testing. Targeting cytochrome C oxidase genes, Kim et al. [19] developed a method to discriminate Korean native honey (KNH) and European honey (EH) using duplex polymerase chain reaction (PCR). However, when we applied these primers on Chinese honey samples, we found that the Korean native honey primers are species-specific, while the European honey primers were not specific to *A. cerana* samples originated from China and the size of the amplified products of the two kinds of bees were the same. Soares et al. [7] designed species-specific primers targeting the tRNAleu-cox2 intergenic region and allowing the detection of *A. cerana* DNA by PCR. With the assistance of high-resolution melting analysis targeting the *16S rRNA* gene, they discriminated *A. cerana* honey from *A. mellifera* honey.

Mitochondrial DNA has been extensively used for phylogenetic and evolutionary relationships analysis among animal species, due to its maternal inheritance, lack of genetic recombination, and rapid evolutionary rate [20]. Mitochondrial genome varies in size from 14 to 19 kb that only contain 36–37 genes among most metazoan species [21,22]. Currently, all genetic testing methods identifying the entomological honey origin were based on mitochondrial DNA. Some genes in the honeybee nuclear genome are highly polymorphic, the *MRJP* gene family is a typical example. Based on the genomic sequencing data in NCBI (National Center for Biotechnology Information), we found that the *MRJPs* (major royal jelly proteins) genes showed certain differences in sequence between *A. cerana* and *A. mellifera*. Since the difference of the *MRJP2* gene sequence between *A. cerana* honey and *A. mellifera* is relatively larger than the other *MRJPs* genes, we hypothesized that the *MRJP2* gene may be used to identify different honey and chose the *MRJP2* gene as our target gene. Multiplex PCR allows discrimination of several species at the same time [23] and real-time PCR with melt curve analysis is a time-saving, cost-effective, and reliable tool to exploit DNA barcoding without additional electrophoresis steps [24]. In this study, the above two methods were used and the aim of our study is to develop a practical, rapid PCR system, for the entomological authentication of honey produced by *A. cerana* and *A. mellifera*.

## 2. Results and Discussion

### 2.1. Selection of PCR Primers

The specificity of the primers was tested experimentally using DNA extracts obtained from honeybee (*n* = 35) and honey (*n* = 72) samples. The PCR products were electrophoresed and the gel was photographed under UV light. Through screening, two pairs of species-specific primers for *A. cerana* honey (C-F, C-R) and *A. mellifera* (M-F, M-R) were selected. The primers’ information is listed in Table 1. 

### 2.2. Specific Detection of *MRJP2* Gene in Honey Using PCR

**Figure 1:**
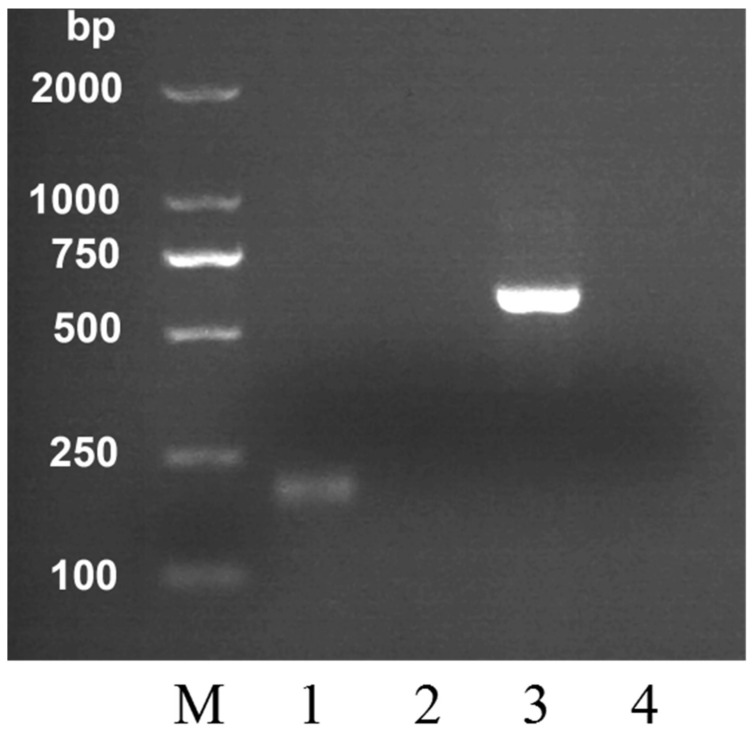
Agarose gel electrophoresis of DNA amplified from genomic DNA extracts of *Apis cerana* honey and *A. mellifera* honey in ordinary PCR with the *A. cerana* honey specific primers (C-F and C-R), and the *A. mellifera* honey specific primers (M-F and M-R). Lane M, DNA marker; Lane 1, C-F and C-R with *A. cerana* honey DNA extracts; lane 2, C-F and C-R with *A. mellifera* honey DNA extracts; lane 3, M-F and M-R with *A. mellifera* honey DNA extracts; lane 4, M-F and M-R with *A. cerana* honey DNA extracts.

After DNA extraction from all analyzed samples, the *MRJP2* gene in honey was detected using the PCR method with selected primers. Amplified DNA products were detected by agarose gel electrophoresis with Gelview staining (Figure 1). The *A. cerana* honey DNA template was used in lane 1 and 4, and *A. mellifera* honey DNA template was used in lane 2 and 3. *Apis cerana* primers were used in lane 1 and 2, while *A. mellifera* primers were used in lane 3 and 4. As shown in Figure 1, DNA templates were only amplified in lane 1 and 3. The primers of *A. cerana* only allowed the detection of *A. cerana* DNA in the corresponding honey samples, while negative amplifications were observed for honeys produced by *A. mellifera* and vice versa, which indicated that our primers had good species specificity. These results were confirmed by all the honey and bee worker samples. The geographical and botanical origin of honeys have no influence on the DNA analysis. After electrophorese, the PCR products were sequenced. The amplified fragments were confirmed as the *MRJP2* gene products of *A. cerana* and *A. mellifera*, respectively. The amplified fragment size of DNA was 212 bp for *A. cerana* and 560 bp for *A. mellifera*. The predicted DNA product size of *A. cerana* and *A. mellifera* was 212 bp and 393 bp, respectively. Interestingly, the PCR amplification product of *A. mellifera* was larger than predicted. In this case, we examined the *MRJP2* gene sequence we obtained and found that the sequence we used to design the primers was the cDNA sequence. The presence of intron fragment may be the reason why the amplified product was larger than the predicted one. The bee DNA in honey may be quite degraded [25], and for this reason the PCR primer pair was designed to amplify fragments of expected size lower than 500 bp. Using our primers, despite degradation of the honey DNA used as template, the PCR was successful in all tested honey samples. These results demonstrated that our species-specific primers could be used to identify the entomological origin of the two kinds of honey, and it is feasible to detect the entomological origin of honey produced by *A. cerana* and *A. mellifera* based on the difference between their *MRJP2* genes.

Ordinary PCR has a wide range of applications. It does not require expensive instrument and its operation is relatively simple. In previous reports, Kim et al. [19] developed a method to discriminate Korean native honey (KNH) and European honey (EH) using duplex PCR. We applied their European honey specific primers on our Chinese honey samples and found that the primers were not specific to *A. cerana* samples originating from China, and the size of the amplified products of the two kinds of bees were the same. With the assistance of high-resolution melting analysis targeting the 16S rRNA gene, Soares et al. [7] successfully discriminated *A. cerana* honey from *A. mellifera* honey. As for ordinary PCR analysis, they only got specific primers for *A. cerana.* Both of the above studies lacked effective *A. mellifera* honey primers that can directly identify *A. mellifera* DNA using an ordinary PCR method. However, without specific *A. mellifera* primers, it may lead to misjudgment in identification. In our research, species-specific primers for *A. mellifera* honey was obtained. The geographical and plant origin of honeys had no influence on the DNA analysis and the results were confirmed by our 72 honey and 35 bee worker samples. Our research made it more practical and feasible to discriminate *A. cerana* honey from *A. mellifera* honey by ordinary PCR method.

### 2.3. Detection Sensitivity and Application in Adulteration

**Figure 2:**
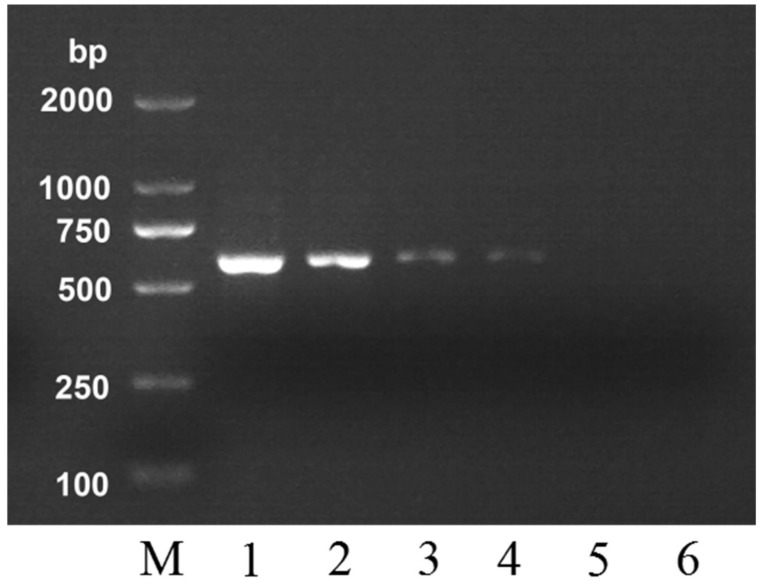
Agarose gel electrophoresis of PCR products using *A. mellifera* honey specific primers with a serial dilution of *A. mellifera* honey DNA extract. Lane M, DNA marker; Lane 1, 100 ng; lane 2, 10 ng; lane 3, 1 ng; lane 4, 0.1 ng; lane 5, 0.01 ng; lane 6, negative control.

To verify the sensitivity of this PCR detection method, a ten-fold serially diluted gradient dilution of DNA extract (100 ng to 0.01 ng) obtained from *A. mellifera* was used. The PCR conditions using M-F/M-R primers allowed achieving a sensitivity of 0.1 ng (Figure 2). The brightness of the characteristic strip of *A. mellifera* honey was gradually dimmed as the amount of the DNA template reduced. The strip can still be observed even when the DNA template amount was as low as 0.1 ng (lane 4), which shows that the sensitivity of this method is very high. As reported by Soares et al. [7], the concentration of DNA extract from honey ranged from 2.3 to 303.9 ng/μL. In our method, the DNA content in honey meets the requirement for the amount of DNA template required for identification.

**Figure 3:**
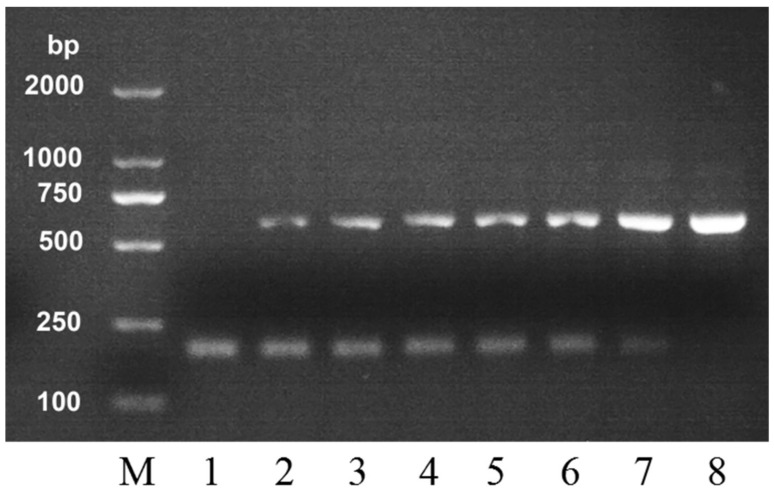
Agarose gel electrophoresis of DNA amplified from genomic DNA extracts of the mixtures of *A. cerana* honey and *A. mellifera* honey in duplex PCR. Lane M, DNA marker. From left to right, the proportion of *A. mellifera* honey was 0, 1, 5, 10, 30, 50, 80, and 100 percent.

In order to further test the detection sensitivity of this PCR method on the adulteration of *A. mellifera* honey, we mixed *A. cerana* honey with *A. mellifera* honey in different proportions and analyzed their DNA. The mixed samples were detected by duplex PCR method, and the result is shown in Figure 3. From left to right, the proportion of *A. mellifera* honey was 0, 1, 5, 10, 30, 50, 80, and 100 percent in the mixture. The species-specific band (560 bp) of *A. mellifera* honey still appeared even when the percentage of *A. mellifera* honey in the mixture was only 1 percent. The brightness of this band deepened with the increase of *A. mellifera* honey. When the proportion of *A. mellifera* honey was 100 percent, the species-specific band (212 bp) of *A. cerana* honey disappeared. Hence, when the DNA amplification products of an *A. cerana* sample shows a band with a molecular weight of 560 bp, the species-specific band of *A. mellifera* honey, it can be reasonably doubted that this *A. cerana* honey was adulterated with *A. mellifera* honey. Therefore, our PCR analysis could be an analytical method to determine the authenticity of entomological origin of the two kinds of honey.

### 2.4. Real-Time PCR Method Development and Melt Curve Analysis

**Figure 4:**
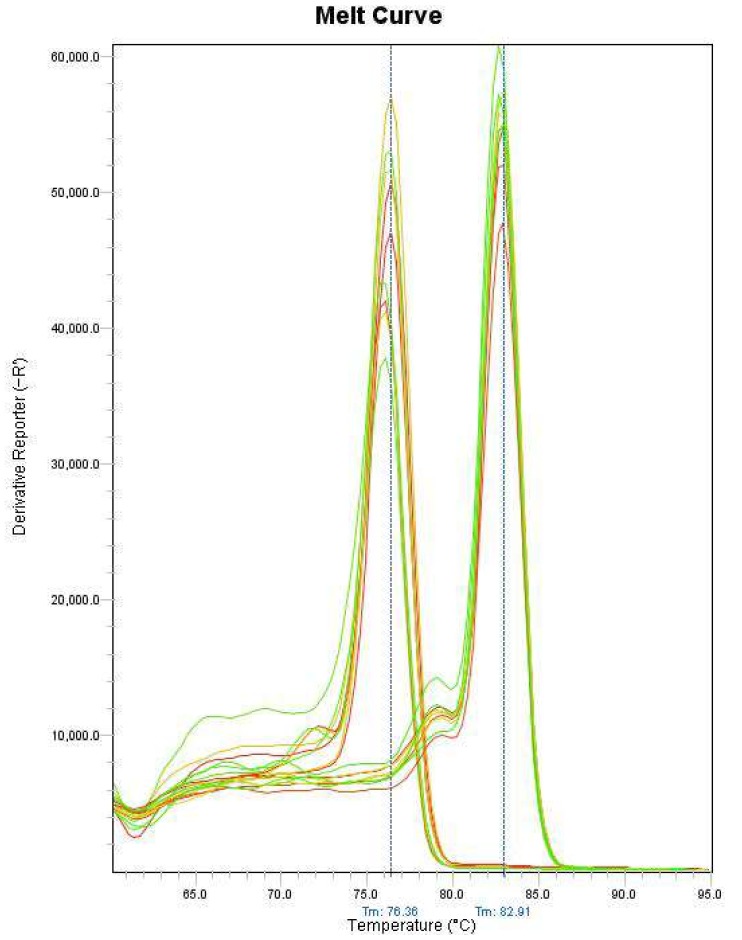
Conventional melting curves obtained by real-time PCR amplification targeting *MRJP2* gene. Left cluster, *A. cerana* honey; right cluster, *A. mellifera* honey.

For more sensitive and quicker detection, a real-time PCR assay was also proposed using the same primers. After real-time PCR amplification, the PCR product was sequenced from both ends. Using BLAST, the amplified fragments were confirmed as the MRJP2 gene products of *A. cerana* and *A. mellifera*. Primers can only amplify DNA templates of corresponding bee species. This result was further confirmed by agarose gel electrophoresis. In addition, the analysis to melt curve of PCR products verified the specificity of amplification (Figure 4). The amplification products exhibited a good resolution, both by melting curve analysis, with single melt peaks for each species (76.4 °C for *A.cerana* and 82.9 °C for *A. mellifera*). The melt curves of the two kinds of honey can be clearly distinguished, suggesting the effectiveness of the technique for the identifying the entomological origin of honey produced by *A. cerana* and *A. mellifera.* The geographical and plant origin of honeys had no influence on the DNA analysis and the results were confirmed by our 72 honey and 35 bee workers samples. Comparing to the duplex PCR method, this real-time PCR assay is a time-saving and more reliable tool to exploit DNA barcoding without additional electrophoresis step.

Our research shows that *A. cerana* honey and *A. mellifera* honey can be identified by the difference in nuclear genes between the two kinds of bee. In addition to the *MRJP2* gene, other nuclear genes may also be used for the identification of honey. Our study extends the possibility of gene selection in identification. *A. cerana* and *A. mellifera* have many subspecies, our samples did not cover all subspecies due to experimental limits. Though our results show a successful identification of *A. cerana* honey and *A. mellifera* honey, further testing is merited to use a statistically significant number of honeys produced by other subspecies under *A. cerana* and *A. mellifera*. Ordinary PCR and real-time PCR each has its own advantages and disadvantages. These two methods we proposed meet the requirements for the entomological authentication of honey samples under different instrument and equipment conditions.

## 3. Materials and Methods

### 3.1. Honey and Honeybee Samples

*Apis cerana* honey samples were provided by beekeepers from two Asian countries, China (*n* = 30) and Vietnam (*n* = 3). *Apis mellifera* honey samples were harvested directly by beekeepers from China (*n* = 31), Brazil (*n* = 3), Australia (*n* = 3), and South Africa (*n* = 2). *Apis cerana* and *A. mellifera* workers were collected from 35 colonies (15 colonies for *A. cerana* and 20 colonies for *A. mellifera*) around China. Ten *A. cerana* samples and 15 *A. mellifera* bee samples were collected from the same colony where honey samples were collected. All samples were collected in 2016–2018. Honey samples were stored in glass bottles at 4 °C and honeybee samples were stored at −80 °C before use.

### 3.2. DNA Extraction

Extraction of DNA from honey samples was carried out following the protocols reported by Utzeri et al. [25] and Soares et al. [7] with some modifications. Briefly, a total of 15 g of honey from each sample was diluted in 50 mL of ultrapure water. Mixture was incubated at 45 °C for 5 min, homogenized and centrifuged at 20,000× *g* for 30 min. The supernatant was discarded afterwards, the pellet was suspended in 1 mL of ultrapure water and again centrifuged at 20,000× *g* for 15 min. Subsequently, the supernatant was discarded and the pellet stored at −80 °C until DNA extraction. 

The DNA pellets from honey samples were extracted using the GMO Food DNA Extraction Kit (Tiangen biochemical technology Co., Ltd., Beijing, China). Total DNA extractions from bees were carried out using TIANamp Genomic DNA Kit (Tiangen biochemical technology Co., Ltd., Beijing, China) following the instructions for animal tissues. The concentrations of DNA samples were determined by Nano Drop spectrophotometer (ND-2000, NanoDrop Technologies, Madison, WI, USA).

### 3.3. Design and Selection of PCR Primers

We chose *MRJP2* gene as the target area. The *MRJP2* gene sequences of *A. cerana* (AY392758.1) and *A. mellifera* (406091) were obtained from NCBI. Primers were designed using Primer Premier 5.0 (Premier Biosoft International, Palo Alto, CA, USA). Primer specificity was assessed by Primer-BLAST tool (http://www.ncbi.nlm.nih.gov/tools/primer-blast). Eight pairs of primers were obtained (4 for *A. mellifera* and 4 for *A. cerana*). Designed primers were synthesized by Sangon Biotech (Shanghai) Co., Ltd. (Shanghai, China).

### 3.4. PCR

Polymerase chain reaction (PCR) amplification was conducted in a 20-µL reaction volume mixture containing 10 µL 2× Taq PCR StarMix with Loading Dye (GenStar BioSolutions Co., Ltd., Beijing, China), 10 μm of each oligonucleotide primer, 2 µL template DNA, and 6 µL double distilled water. For duplex PCR, two pairs of primers were added and the amount of distilled water was reduced to 4 µL; other ingredients remained unchanged. The thermal cycling steps consisted of an initial pre-denaturation at 94 °C for 2 min, followed by 35 cycles of denature at 94 °C for 30 s, 55 °C for 30 s, 72 °C for 30 s, and a final extension of 72 °C for 5 min. The PCR amplification was carried out in a T-Gradient Thermoblock (Biometra, Göttingen, Germany). The PCR products (7 µL per reaction) were electrophoresed in 2% Tris-acetate-EDTA–agarose (Biowest, Shanghai, China) gel containing 0.01% Gelview (BioTeke, Beijing, China). The agarose gel was visualized and photographed under UV light using automatic gel imaging analyzer (JS-6800, Shanghai Peiqing Science & Technology Co., Ltd., Shanghai, China). The positive products were recycled and then sequenced from both ends using an ABI 3730XL automatic sequencing system (TSINGKE, Hangzhou, China). Sequence specificity analyses for the *MRJP2* fragment were performed using BLAST (http://blast.ncbi.nlm.nih.gov/) (National Library of Medicine, Bethesda, MD, USA).

### 3.5. Real-Time PCR and Melt Curve Analysis

The real-time PCR was conducted using StepOne plus (Applied Biosystems, Carlsbad, CA, USA) with SYBR Green Ex Taq (TaKaRa, Dalian, China) in 20 μL of total reaction mixture. The primers used were the same as ordinary PCR. The following conditions were used for amplification: 95 °C for 30 s, 40 cycles at 95 °C for 5 s, and 60 °C for 30 s.

For melt curve analysis, real-time PCR products were denatured at 95 °C for 15 s and then annealed at 60 °C for 1 min to allow the correct annealing of the DNA duplexes. These two steps were followed by melting curve ranging from 60 to 95 °C with temperature increments of 0.3 °C every 10 s. The data of real-time PCR and melt curve analysis were processed using StepOne Software v2.3 (Applied Biosystems, Carlsbad, CA, USA).

## 4. Conclusions

In the present work, we proposed a DNA-based assay to detect the entomological origin of honey produced by *A. cerana* and *A. mellifera* based on the *MRJP2* gene of the two kinds of bee. The duplex PCR method can detect even one percent *A. mellifera* honey incorporation in *A. cerana* honey. The melt curves of the two kinds of honey can be clearly distinguished, suggesting the effectiveness of the real-time PCR method. These two methods we proposed could satisfy different needs for entomological authentication of honey samples under different instrument and equipment conditions. This study is beneficial to the exploitation of *A. cerana* honey resources and also to the development of *A. cerana* breeding industry. Our research may also provide a reference for the discrimination of honeys produced by other bee species.

## Figures and Tables

**Table 1:** Primers used for polymerase chain reaction (PCR) amplification.

Honey	Primer	DNA Sequences	Product Size (bp)
*A. cerana*	C-F	TTTAACAATAAAAATAATCAGAAGA	212
	C-R	TTACATCCTAATTGATTTTAATGCG	
*A. mellifera*	M-F	GCCATCCCTTGAAATTGTCACTCGT	560
	M-R	TCTGCAAACGACCAATCAGGATAT	
